# Noncoding RNAs in Unexplained Recurrent Spontaneous Abortions and Their Diagnostic Potential

**DOI:** 10.1155/2019/7090767

**Published:** 2019-12-04

**Authors:** Ningyi Jia, Jian Li

**Affiliations:** Beijing Obstetrics and Gynecology Hospital, Capital Medical University, Beijing, China

## Abstract

Unexplained recurrent spontaneous abortion (URSA) is defined as the loss of two or more consecutive pregnancies before the 20th week of gestation with normal findings on routine screening tests. Our understanding of the cellular and molecular pathogenesis of URSA is still far from complete. Noncoding RNAs (ncRNAs) play a pivotal role in transcription and expression. The functions of ncRNAs may also improve understanding of URSA pathogenesis. Because of their stability in the circulatory system and at the maternal-fetal interface, it may be possible to use ncRNAs as biomarkers for certain disease states. Here, we provide a narrative review of the current state of knowledge about ncRNAs associated with URSA. The possibility of developing a diagnostic tool using ncRNAs is discussed. The underlying mechanisms of how ncRNAs may lead to the onset of URSA are explored in this review.

## 1. Introduction

Unexplained recurrent spontaneous abortion (URSA) is defined as the loss of two or more consecutive pregnancies before the 20th week of gestation with normal findings on routine screening tests (such as normal parental karyotypes, endocrine, infection, and immune parameters) [1]. URSA accounts for 15% of recurrent spontaneous abortions [2]. The etiology of URSA remains poorly defined owing to it being identified by a diagnosis of exclusion and its complex molecular and cellular regulation. It is suggested that aberrant gene expression is a major cause of URSA and an important indicator of pregnancy disorders [3, 4]. ncRNAs are important regulators of transcription and protein expression [5]. The dysregulation of ncRNAs may be involved in the etiology of many pregnancy-related disorders, such as URSA. In biomedical research, ncRNAs are gaining increasingly greater importance as novel biomarkers for diagnosis, prediction, prognosis, and reaction to therapy in diseases. Apparently, ncRNA expression profiling is more effective than mRNA expression profiling for differentiating between normal and pathological tissues [6]. Therefore, the link between URSA and ncRNAs may offer new insights into the pathogenesis and clinical diagnosis of this disease.

Noncoding RNA (ncRNA) can be classified based on its length. One class consists of small transcripts less than 200 nucleotides long. (This class includes microRNAs (miRNAs).) The other class consists of long noncoding RNAs (lncRNAs), which have a length of no less than 200 nucleotides [7]. There are some other kinds of ncRNAs such as circular RNA (circRNA), small nucleolar RNA (snoRNA), piwi-interactive RNA (piRNA), and small nuclear RNA (snRNA) [8]. Considering the wide range of findings published on miRNA and lncRNA, we focus on these two ncRNAs in our review.

**Figure 1:**
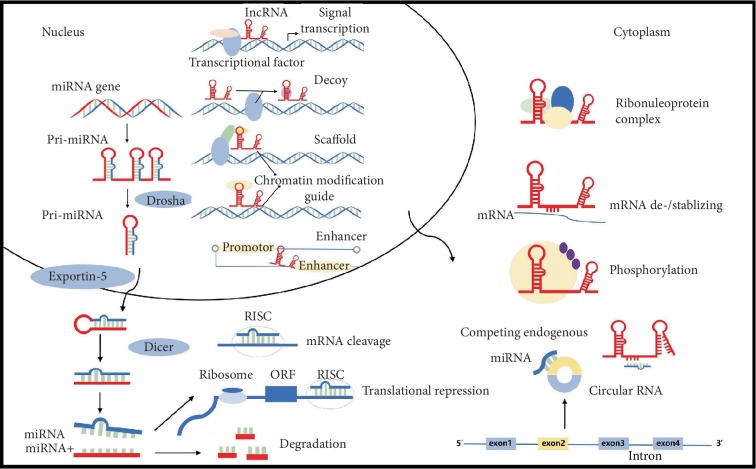
The functions of miRNA and lncRNA and the interaction of ncRNAs. Pre-miRNA is transported into the cytoplasm through exportin-5 and Ran-GTP where it is cleaved by Dicer and finally converted into mature miRNA. This double-stranded small RNA separates into two single strands. One strand from mature miRNA is loaded into the RISC that recruits target mRNA and initiates either translation inhibition or mRNA degradation. lncRNA acts as scaffolds, signals, decoys, guides, miRNA sponges, and enhancer RNAs. They can also contribute to the stability of mRNA. They can serve as downstream regulators of miRNA by acting as competing endogenous RNAs. circRNAs act as miRNA sponges or competing endogenous RNA.

Each type of ncRNA has distinct roles in various physiological and pathophysiological processes. With a length of 18-25 nucleotides, miRNA is the dynamic regulator of gene expression [9]. miRNAs perform their functions as part of the RNA-induced silencing complex, which is capable of recruiting target mRNA and initiating translation inhibition or mRNA degradation [10] (Figure 1). CircRNAs can serve as miRNA sponges by binding to proteins to regulate the expression of target genes at the transcriptional level [11]. In recent years, reports have shown that lncRNAs play an important role in chromatin modification, transcription, translation, gene imprinting, regulation of protein bioactivity, and alternative splicing [12, 13]. They act as scaffolds, signals, decoys, guides, miRNA sponges, and enhancer RNAs. Proteins and other RNAs can be bound by scaffold lncRNAs to form larger functional complexes. lncRNAs can act as signals to regulate transcription. Decoy lncRNAs are able to block the normal function of target proteins by binding to specific domains. They can also serve as downstream regulators of miRNA by acting as competing endogenous RNAs [14, 15]. In addition, small regulatory RNAs such as miRNAs can be bound and sequestered by lncRNAs [16], while enhancer RNAs play an important role in the regulation of the expression of neighboring genes. These ncRNAs may have clinical and pathological connections with human diseases.

The genesis and development of the placenta are closely related to the proliferation, differentiation, and functional status of trophoblast cells. It is generally believed that trophoblast cell dysfunction mediates pregnancy disorders [17]. Previous studies have shown that ncRNAs are involved in several steps of the implantation process. Certain ncRNAs have been shown to significantly contribute to the dysfunction of trophoblasts in pregnancy-related disorders [18–21]. Moreover, many publications have reported that the failure of maternal-fetal immunological tolerance and responses can contribute to the pathogenesis of URSA [22–25]. Therefore, identifying certain molecules, as well as their roles in the maternal-fetal interface and peripheral blood, will enable us to have a more comprehensive understanding of the pathogenesis of URSA. The abnormal expression of certain ncRNAs may provide new insights into potential biomarkers for URSA. We focus on ncRNAs in URSA patients in this review.

## 2. ncRNA Expression Profiling in URSA

Abnormal expression of ncRNA at the maternal-fetal interface has been observed in URSA (Table 1). To measure differential expression of ncRNA in a specific morbid state, a widely used approach is genome-wide ncRNA profiling by either microarray or RNA-sequencing, followed by reverse transcription quantitative PCR (RT-PCR) [26]. Since ncRNAs are involved in many biological processes, the change in the level of ncRNAs at the maternal-fetal interface may be associated with URSA pathogenesis.

Dong et al. used a miRNA microarray to profile differentially expressed miRNAs in villi and decidual cells isolated from a URSA sample and a control sample. A total of 50 miRNAs were found to be differentially expressed in villi, and seven miRNAs were found upregulated in the decidua. Gene ontology (GO) analysis showed that the most significantly expressed miRNAs with a fold change of greater than 5 or less than 0.2 were involved in several biological processes, such as regulation of transcription and protein amino acid phosphorylation, whereas a Kyoto Encyclopedia of Genes and Genomes (KEGG) pathway analysis indicated that the predicted target genes were linked to adherence junctions, apoptosis, and the T cell receptor signaling pathway [27]. Specifically, according to the KEGG pathway analysis, the target genes of those miRNAs were deeply involved in the mitogen-activated protein kinase (MAPK) pathway, B cell receptor, and T cell receptor signaling pathways during the development of URSA [28]. The MAPK signaling pathway is of great importance in maintaining normal pregnancy processes, including embryonic development, differentiation of trophoblast cells, and vascular endothelial cell proliferation and differentiation [29, 30]. Two more studies also illustrated the relationships between target genes and signaling pathways [31, 32]. Wang et al. reported that predicted target genes participate in programmed cell death, apoptosis, and cell proliferation in the decidua. The major pathways included ErbB signaling, focal adhesion, p53-signaling, and apoptosis. In villi, target genes are widely involved in the regulation of cell proliferation, apoptosis, and angiogenesis. The KEGG pathway analysis revealed that the relevant pathways were apoptosis, p53-signaling, and the cell cycle [32].

A study on genome-wide differential expression of lncRNA showed that, predominantly, infection and inflammation pathways are the ones altered in URSA [33]. Wang et al. imply that the differentially expressed lncRNAs are involved in pathways related to immunity. Furthermore, lncRNA-regulated genes are believed to be important regulators of immune cell activation and differentiation. It is suggested that lncRNAs contribute to the pathogenesis of URSA through immunity pathways, which might represent a new insight to the relationship between immunity and URSA [34].

CircRNAs are a recently discovered group of ncRNA, characterized by a circular covalently closed structure. The functions of circRNAs include acting as miRNA sponges or competing with endogenous RNA, regulating gene transcription, and interacting with RNA-binding proteins (RBP) [11]. The sequestration of miRNA-binding sites by circRNA weakens the inhibitory effects of miRNAs on gene expression. A circRNA microarray analysis identified aberrantly expressed circRNAs in the villi of patients with URSA. This may shed light on the role of circRNAs in the onset of URSA [35]. The circRNA microarray revealed miRNA-binding sites on many circRNAs. Downregulation of the miRNAs miR-520f [27] and miR-181d [28], which have relevant binding sites on upregulated circRNAs, in URSA dNK cells may contribute to the inhibition of adhesion and cell proliferation [35]. However, the roles of circular RNA and competing endogenous RNA networks remain to be investigated.

The above-mentioned studies strongly suggest that ncRNA dysregulation is closely linked to URSA, in which differentially expressed ncRNAs might play a role in pathogenesis.

## 3. Structural Changes in ncRNAs in URSA

The change of ncRNA structure may also contribute to the pathogenesis of URSA. It is believed that single nucleotide polymorphisms (SNPs) in miRNAs could influence biological processes by changing miRNA target selection and contribute to the occurrence of diseases [36]. It has been reported that the miRNA miR-423 may be implicated in the genetic predisposition to URSA because it disrupts the production of mature miR-423 and its target PA2G4 [37]. Furthermore, the miRNA miR-10a impairs the expression of Bim, which affects URSA progression [38]. Also, in the coding region of miR-323b, the SNP rs56103835 T>C was associated with URSA. The expression of miR-323b was elevated in cell lines transfected with the T-C haplotype. The T-C haplotype can inhibit proliferation and migration and promote apoptosis in HTR-8/SVneo cells [39]. One nucleotide mutation in the coding region of pri-miR-125a was associated with the onset of URSA, which was due to two polymorphisms found in patients. The A>G mutation reduced miR-125a expression. Mutant pri-miR-125a can enhance endometrial stromal cell (ESC) invasiveness and disturb the expression of the miR-125a targetome, which influences cell proliferation, migration, invasion, and embryonic development [40, 41]. It was suggested that miR-196a2CC, miR-499AG+GG, and the miR-196a2CC/miR-499AG+GG combination are associated with URSA [42]. The variants of miR-27a also play a pivotal role in predicting folate levels in URSA patients [43].

## 4. ncRNA May Contribute to the Pathogenesis of URSA

Studies suggested that abnormal expression of ncRNAs can result in disorders of pregnancy. Wang et al. [44] provide evidence that miR-133a was strongly overexpressed in URSA villi. The following findings confirm that miR-133a decreases expression of histocompatibility antigen, class I, G (HLA-G), which can lead to impairment of decidual NK cell invasion and angiogenesis [45]. The change of miR-27a-3p, miR-29a-3p, miR-100-5p, miR-127-3p, and miR-486-5p levels in villi can lead to the abnormity of invasiveness in URSA [46]. In another study, it was found that overexpressed miR-520 in the villi of URSA patients was correlated with trophoblast cell apoptosis via suppression of PARP1 expression [47]. Zhu et al. found that miR-16 expression was significantly upregulated in the decidua and villi of URSA patients. It was suggested that miR-16 regulates placental angiogenesis by influencing VEGF expression, which may contribute to the pathogenesis of URSA [48]. It has been verified that compared to normal mice, miR-3074-5p expression was significantly upregulated and miR-486-3p expression was significantly downregulated in mice with URSA [49]. The later research provided evidence that miR-3074-5p promotes the apoptosis but inhibits the invasiveness of HTR8/SVneo cells in vitro [50].

Studies show that the lncRNA HOTAIR can be inhibited in URSA, which can influence trophoblast invasion and the expression of Matrix Metallopeptidase 9 (MMP9) [51, 52]. Moreover, Lnc-SLC4A1-1 can recruit nuclear factor kappa-light-chain-enhancer of activated B cells (NF-*κ*B) and ultimately upregulate the expression of interleukin 8 (CXCL8). This in turn elevates the levels of tumor necrosis factor alpha (TNF-*α*) and interleukin 1 beta (IL-1*β*) and affects trophoblast function. Dysfunction of Lnc-SLC4A1-1 may affect immune response and lead to URSA [53].

**Figure 2:**
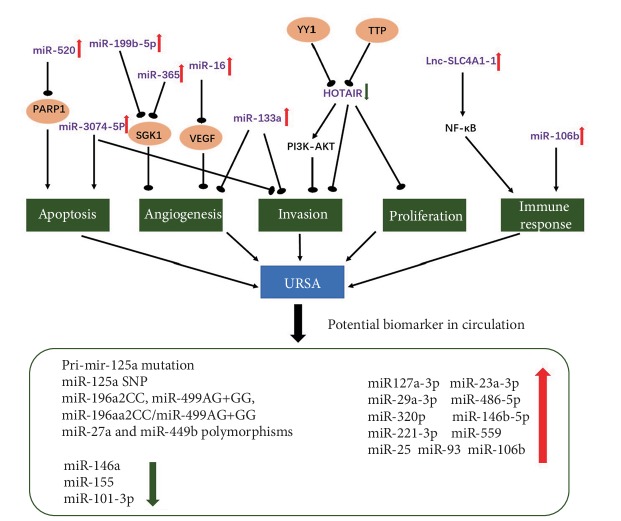
The dysregulation of ncRNAs and their function in the pathogenesis of URSA. The potential diagnostic ncRNAs in circulation in URSA patients.

Researchers have found that miRNAs can influence the balances of TH1/TH2 and TH17/Treg cells in many pregnancy complications [54, 55]. Recent studies reveal that miRNAs participate in the establishment of immune tolerance at conception and may contribute to the regulation of dendritic cells and T regulatory cells generated by seminal fluid [56]. The miR-106b-25~93 cluster, recognized as Treg-associated miRNAs, showed a higher expression in URSA patients. An imbalanced immune response and dysregulated T cell function may lead to reproductive failure. Immune cell-related miRNA profiles may serve as biomarkers and be used for making prognoses during treatment of URSA patients [55]. As is mentioned above, miR133a in the villi can lead to downregulation of HLA-G expression and impairment of angiogenesis and invasion [45]. These immune abnormalities generate an inflammatory response, thus affecting function at the maternal-fetal interface (Figure 2).

## 5. ncRNA as a Potential Diagnostic Tool for URSA

Although it has been verified that ncRNA expression profiles are different in different disease states, obtaining biopsy samples from organs for pathological profiling is unrealistic. Samples obtained from the circulation offer an ideal alternative. The majority of circulating miRNAs are associated with protein complexes or high-density lipoprotein [57, 58]. Meanwhile, miRNAs may also be found encapsulated within cellular vesicles [59, 60]. The “packaging” of miRNAs contributes to their stability in plasma. Owing to their stability in blood, ncRNAs have been explored as biomarkers for many disease states. The differential expression of ncRNA has been reported in cancers [61], cardiac disease [6], and some pregnancy disorders such as preeclampsia [62–64]. The predictive potential of these ncRNAs as early indicators of disease is highlighted in this review.

Published studies have reported abnormal expression of some miRNAs in maternal circulation. Although these studies put forth the idea that these circulating miRNAs might be biomarkers of URSA, in some of them, a receiver operating characteristic (ROC) curve has not been performed and the results need to be validated in an independent cohort. Yang et al. suggest that levels of miR-127a-3p and miR-486-5p in plasma could serve as predictive factors for URSA [46]. Qin et al. found four circulating miRNAs that were upregulated and one circulating miRNA that was downregulated that demonstrated potential as biomarkers for URSA [65]. miRNAs play a pivotal role in peripheral blood monocular cells (PBMCs), in regulation and differentiation of T cells. In PBMCs, miR-25, miR-93, and miR-106b were found to be elevated while miR-146-a and miR-155 were downregulated [55]. Several studies have found that in maternal circulation, mutated miRNAs may be associated with URSA. However, the use of ncRNAs as noninvasive diagnostic biomarkers has not yet been established (Figure 2). Thus, studies on larger populations are needed in order to validate the potential of ncRNAs.

ncRNAs could potentially be used for the treatment of pregnancy complications in the future. A single ncRNA may affect multiple cellular pathways. ncRNAs are shed from primary cells into the circulation and are involved in cell-to-cell communication. The targeting of a single ncRNA may affect multiple downstream pathways. Recently, many different methods of silencing lncRNAs in cancer have been reported. However, targeting pathways may result in some side effects. From what has been discussed above, the functions of ncRNAs should be better understood before they are used as therapeutic targets.

There are no commonly accepted methods for diagnosis of URSA nor any efficient treatments, due to the lack of studies with abundant sample size, independent validation cohorts, or analysis of correlation with disease characteristics. Major gaps in knowledge need to be filled before findings can be translated into clinical applications.

## 6. Conclusion

URSA is not a rare disease, but its molecular and cellular pathogenesis remains poorly understood. Emerging evidence shows that the expression profiles of ncRNAs at the maternal-fetal interface and in the circulation of URSA patients have a biological relevance, allowing for a better understanding of the disease. Through microarray and RT-PCR assays, some dysregulated ncRNAs in both URSA cells and tissues have been identified. A growing number of studies have suggested functional roles for ncRNAs in URSA and provided new clues for their clinical application as potential biomarkers. However, functional studies of these ncRNAs are still limited. Existing research can provide us some insight into the pathogenesis of URSA.

Although many studies have demonstrated that ncRNAs are critical in placental development, differences exist between animals and humans in placental development and placental ncRNA expression profiles. Many studies of ncRNAs in placenta were performed using cell lines, which involve different conditions compared to experiments performed in vivo. The roles of ncRNAs in the pathogenesis of URSA are still far from being understood, especially when compared with the progress made in other disease fields. Further investigation of the contributions of ncRNAs for modulating cellular activities in URSA is required.

## Figures and Tables

**Table 1:** ncRNA expression profiles in the maternal-fetal interface of URSA.

Reference	Microarray filtering criteria	Method	Sample	Upregulated	Downregulated
[27]	*q* value ≤ 0.05; FC ≥ 2 or ≤0.5	Microarray qRT-PCR	Villi Decidua	4 miRNAs 7 miRNAs	41 miRNAs Not mentioned
[28]	FC ≥ 2.0; *p* value ≤ 0.05	Microarray qRT-PCR	Villi	98 miRNAs	57 miRNAs
[31]	FDR < 0.05; *p* value < 0.005	Microarray	Decidua	49 miRNAs	1 miRNA
[32]	FC > 2.0; *p* value < 0.05	RNA-seq qRT-PCR	Decidua Villi	23 miRNAs 4 miRNAs	Not mentioned 5 miRNAs
[33]	FC > 2.0	Microarray qRT-PCR	Decidua	859 lncRNAs	940 lncRNAs
[34]	FC > 2.0	Microarray qRT-PCR	Villi	467 lncRNAs	982 lncRNAs
[35]	FC ≥ 2.0; *p* value ≤ 0.05	Microarray qRT-PCR	Villi	335 circRNAs	259 circRNAs

FC: fold change; FDR: false discovery rate.
